# Facile Synthesis of 3D Printed Tailored Electrode for 3-Monochloropropane-1,2-Diol (3-MCPD) Sensing

**DOI:** 10.3390/mi13030383

**Published:** 2022-02-27

**Authors:** Farrah Aida Arris, Denesh Mohan, Mohd Shaiful Sajab

**Affiliations:** 1 Research Center for Sustainable Process Technology (CESPRO), Faculty of Engineering and Built Environment, Universiti Kebangsaan Malaysia, Bangi 43600, Selangor, Malaysia; farrahaidaarris@gmail.com (F.A.A.); denesh.mohan@gmail.com (D.M.); 2 Department of Chemical and Process Engineering, Faculty of Engineering and Built Environment, Universiti Kebangsaan Malaysia, Bangi 43600, Selangor, Malaysia

**Keywords:** additive manufacturing, electrodeposition, palm oil contaminant, cyclic voltammetry, 3-monochloropropane-1,2-diol (3-MCPD), zero-valent iron (ZVI)

## Abstract

Additive manufacturing (AM) has allowed enormous advancement in technology and material development; thus, it requires attention in developing functionalized printed materials. AM can assist in efficiently manufacturing complex tailored electrodes for electrochemical sensing in the food industry. Herein, we used a commercial fused deposition modeling (FDM) filament of acrylonitrile butadiene styrene (ABS) for FDM 3D printing of a self-designed electrode with minimal time and cost compared to a commercial electrode. A graphene-based ABS conductive filament (ABS-G) was used to fabricate the conductive electrode in a dual-nozzle FDM 3D printer. The electrochemically conductive 3D printed electrode was characterized using cyclic voltammetry and tested against standard 3-monochloropropane-1,2-diol (3-MCPD) with known concentrations using an amperometric detection method. Results showed a basis for promising application to detect and quantify 3-MCPD, a food contaminant known for its carcinogenic potential. The fabrication of functionalized 3D printed polymer electrodes paves way for the development of complete 3D printable electrochemical sensors. Under optimal conditions, this newly synthesized electrochemical sensor exhibited sensitivity with a linear response range from 6.61 × 10^−4^ to 2.30 × 10^−3^ µg/mL with an estimated limit of detection of 3.30 × 10^−4^ µg/mL against 3-MCPD.

## 1. Introduction

AM technology is developing rapidly with various types of AM techniques due to the ease of product manufacturing and production of customized products [1,2,3]. FDM is the most common AM technology used for fabrication because of the inexpensive FDM 3D printer and materials [4]. The ABS material for FDM 3D printing has been extensively evaluated for polymer-based applications, but its use has been limited in electrochemical sensing applications due to its electrical insulating properties [5]. The advancement in FDM 3D printing polymer modification with conductive fillers such as graphene and carbon black could pave way for the fabrication of customized portable and complex sensing devices [6,7,8,9]. Further functionalization of the conductive 3D printed electrodes with gold (Au) and silver chloride (AgCl) has been achieved for commercial electrochemical sensing [6,10,11]. This development could be further investigated for sensor application in the food industry with functionalized materials for the detection of various types of analytes such as mycotoxins and 3-monochloropropane-1,2-diol (3-MCPD) [12].

In 2016, the European Food Safety Authority (EFSA) started to evaluate the severity of consuming foods containing 3-MCPD, which is an organic chemical compound found in processed foods such as refined vegetable oils, breads, and soy sauce [13]. The glycerol-based contaminant was concluded to be potentially carcinogenic according to toxicological data from tests performed on rats, imposing danger to humans upon consumption beyond tolerable limits [14]. Among refined vegetable oils, palm oil was reported to have the highest 3-MCPD content [15,16,17]. The formation of 3-MCPD was contributed by the presence of several precursors such as acylglycerols and chloride-containing compounds, with the combination of other factors such as conditions during the oil refining process, which applies elevated temperatures up to 260 °C and the addition of acid-activated bleaching during the deodorization stage [16,17,18,19,20,21,22,23].

Due to the potential risk of 3-MCPD, a safe intake limit was introduced to ensure that palm oil is safe for human consumption. The intake limit of 3-MCPD was set to 2 µg/kg body weight per day by the FAO/WHO Expert Committee on Food Additives, while a 0.02 mg/kg regulatory limit was set for hydrolyzed vegetable protein and soy sauce by the European Union [24]. To comply with the 3-MCPD safe limit, reliable methods of detection and quantification must be standardized and used. The most frequently used method to detect and quantify 3-MCPD content is gas chromatography–mass spectrometry (GC–MS). GC–MS, however, requires the oil sample to be prepared by a derivatization process for the 3-MCPD to be readable. The oil sample preparation also requires a significant amount of solvent to allow for liquid extraction. GC–MS also needs to be conducted in the laboratory due to the mobility limitation of the instrument and requires skilled personnel to perform the test [25]. On average, GC–MS results can be produced in at least 2 h; therefore, it is considered a time-consuming method. The cost of GC–MS is also not cheap; thus, with the increasing demand to test more frequently for accuracy, it is not economical. Therefore, there is a need to establish a simple, rapid, mobile, and reliable analytical method to quantify 3-MCPD, especially for on-site measurement. One approach is to produce a portable electrochemical sensing device that is able to detect and measure the occurrence of 3-MPCD, as well as the amount at any point during the refining process.

Zero-valent iron (ZVI) is often referred to as an environmentally friendly material, which has been used extensively in wastewater treatment to remediate and remove highly toxic elements such as arsenic(III) in groundwater and supercapacitor electrodes [26,27]. ZVI is also effective for the degradation of halogenated solvents such as chlorinated compounds and is suspected to be able to react with chlorinated solvents, a characteristic that might be useful for targeting contaminated palm oil [28]. The electrochemical deposition method is used to coat or deposit nanomaterials in specific suspensions or solutions by applying the concept of donating electrons to ions [29]. The selection of the electrodeposition method can help to ensure the further reduction of iron in suspension and provide enhanced adhesion of ZVI onto the 3D printed electrode substrate. In this study, a 3D printed acrylonitrile butadiene styrene (ABS)-insulated conductive graphene (ABS-G) electrode was fabricated using a commercial FDM filament. ZVI was tested for deposition onto the newly fabricated 3D printed ABS-G electrode using the drop-casting method, followed by electrodeposition to increase and improve the ZVI adhesion. Next, the 3D printed ABS-G was electrochemically analyzed and calibrated to determine its feasibility for detecting 3-MCPD.

## 2. Materials and Methods

### 2.1. Chemicals and Reagents

Commercially available ABS green filament and ABS conductive filament (graphene-based) of 1.75 mm were procured for FDM 3D printing. Iron(II) sulfate heptahydrate (FeSO_4_·7H_2_O), sodium borohydride (NaBH_4_), potassium chloride (KCl), potassium ferricyanide (K_3_[Fe(CN)_6_]), sodium hydroxide (NaOH), and 3-MCPD were purchased from Sigma-Aldrich (M) Sdn. Bhd. (Selangor, Malaysia). All analytical reagents used in this work were prepared without further refinement and used as obtained. Deionized (DI) water was used as solvent throughout this work.

### 2.2. Instrumentation and Methods

Electrochemical characterization of the newly fabricated 3D printed ABS-G electrode was performed using cyclic voltammetry (CV) on a 910 PSTAT Mini (Metrohm, Herisau, Switzerland) and a three-electrode configuration consisting of the newly 3D printed ABS-G as the working electrode (WE), platinum (Pt) as the counter electrode (CE), and silver/silver chloride (Ag/AgCl) as the reference electrode (RE) in 0.05 M ferricyanide solution as the electrolyte. The electrochemical characterization was performed in order to understand the electron-transfer capabilities of the sensor.

Field-emission scanning electron microscopy (FE-SEM) with energy-dispersive X-rays spectroscopy (EDX) (Merlin Compact, Zeiss Pvt. Ltd., Oberkochen, Germany) was used to characterize the surface morphology and to provide elemental analysis of the deposited ZVI onto the 3D printed ABS-G electrode. The functional groups of the 3D printed electrode were determined using attenuated total reflectance Fourier-transform infrared (ATR-FTIR) spectroscopy (ALPHA FTIR Spectrometer, Bruker, Billerica, MA, USA) in the range of 4000 to 500 cm^−1^ at a resolution of 1 cm^−1^.

### 2.3. Fabrication of 3D Printed ABS-G Electrode

The desired electrode design was modeled using computer-aided design (CAD) software and sliced to a G-code file through slicer software (Ultimaker Cura 4.7, Geldermalsen, Netherlands). The composite electrodes were then 3D printed using a three-axis FDM 3D printer with a dual nozzle (Flashforge Creator 3, Jinhua, China). The printing profile was fixed on the basis of findings from a previous study (nozzle diameter: 0.4 mm, printing temperature: 255 °C, layer height: 0.15 mm; infill percentage: 80%; infill pattern: 45° lines; print speed: 60 mm/s) [30].

### 2.4. Preparation and Deposition of Zero-Valent Iron (ZVI)

Zero-valent iron (ZVI) was synthesized according to [31] with minor modification. FeSO_4_·7H_2_O was diluted in ethanol and DI water, and then reduced using NaBH_4_ to form a greenish-black suspension of ZVI. Deposition of ZVI was performed by drop casting 50 µL of ZVI suspension onto the 3D printed ABS-G electrode surface, which was allowed to dry. Next, the modified 3D printed ABS-G electrode was electrochemically treated and deposited in ZVI suspension using CV mode for five scan cycles to further assist in the reduction of ZVI, as well as the adhesion of nanomaterial onto the electrode surface.

### 2.5. Electrochemical Characterization and Calibration of ABS-G and ABS-G/ZVI

In order to better understand the electroactive capability of the bare and modified electrode to transfer electrons, CV was performed in 0.05 M of K_3_[Fe(CN)_6_] as an electrochemical redox solution using a three-electrode configuration. Calibration against a known 3-MCPD standard solution was executed using amperometric detection in order to understand the feasibility of the sensor to detect and quantify 3-MCPD. The method was performed by adding a fixed volume of 3-MCPD standard in DI water within a fixed interval of time, and the current change was observed over time as a response to the spiked concentration of 3-MCPD in the sample. A scan rate of 100 mV/s was used for the CV experiment unless otherwise stated.

## 3. Results and Discussion

### 3.1. 3D Printing of Electrochemically Conductive Electrode

**Figure 1:**
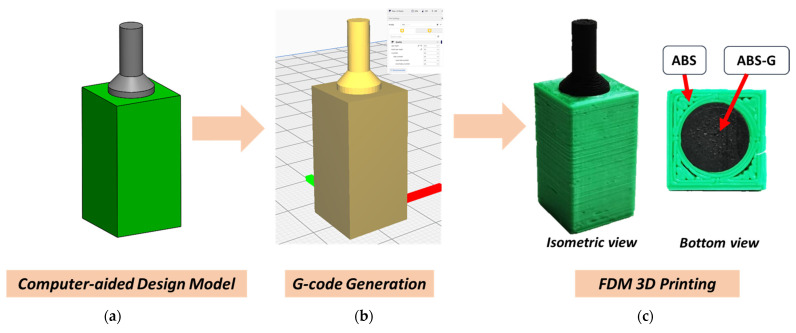
Fabrication process of 3D printed composite electrode. (**a**) Computer-aided design model, (**b**) G-code generation, (**c**) FDM 3D printing.

CAD software was utilized in designing an electrically insulated electrode with ABS in the outer layer, which can be seen in Figure 1a. The G-code comprising an optimized printing profile compatible with a dual-nozzle 3D printer (see Figure 1b) was obtained for FDM 3D printing. The dual-material 3D printed electrode shown in Figure 1c could be fabricated within a short time at low cost, showing the potential of 3D printing for mass commercialization. The automated printing process with high precision using a 0.4 mm diameter nozzle was able to print the dual-material electrode within 58 min. Further development of a conductive filament with higher conductivity will further enhance the development of 3D printed electronics, as the filament can be used directly in the fabrication of electronic circuits, capacitors, and transducers. 

### 3.2. Characterization of 3D Printed ABS-G and ABS-G/ZVI Electrodes

**Figure 2:**
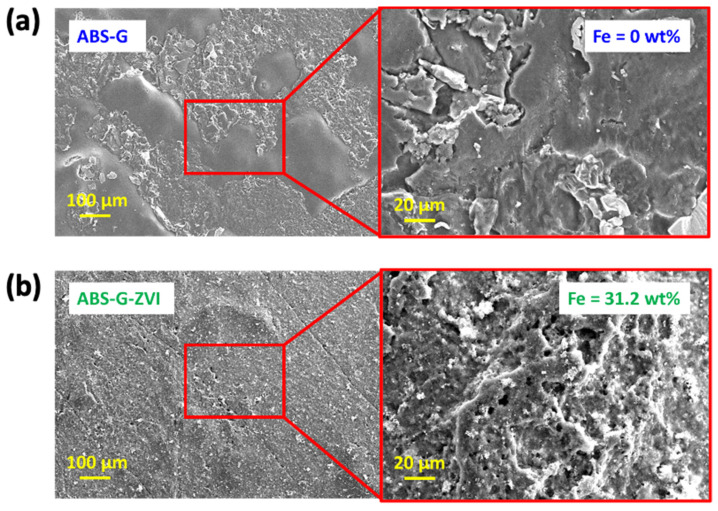
(**a**) Field-emission scanning electron microscope (FE-SEM) and EDX images of the 3D printed ABS-G electrode; (**b**) FE-SEM and EDX images of the 3D printed ABS-G electrode with electrochemically deposited ZVI.

Figure 2a presents the FE-SEM image of the 3D printed ABS-G electrode morphological surface, where the absence of iron nanoparticles was observed. Figure 2b, on the other hand, presents the FE-SEM image of the 3D printed ABS-G electrode with ZVI deposited onto its surface, which can be seen from the agglomeration of iron nanoparticles. Further elemental composition analysis using EDX showed an estimation of 31.2 wt.% of iron nanoparticles successfully deposited onto the 3D printed ABS-G electrode. The improvement in surface roughness after ZVI deposition can also enable better adhesion of nanomaterials to improve analyte detection. The deposited ZVI can also enhance the surface area-to-volume ratio for the available sites to perform electrochemical detection of 3-MCPD.

**Figure 3:**
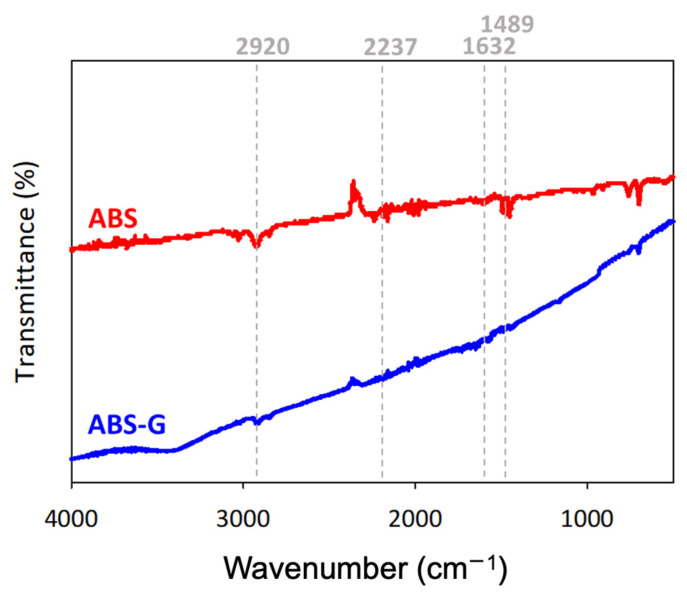
Fourier-transform infrared (FTIR) of 3D printed filaments.

Figure 3 shows the FTIR spectra for the ABS and ABS-G electrode. The FTIR spectrum of ABS (see Figure 3) shows aliphatic stretching at 2920 cm^−1^, acrylonitrile at 2237 cm^−1^, C=C of butadiene at 1632 cm^−1^, and the styrene unit at 1489 cm^−1^ [12]. The graphene content in the ABS-G filament, reported to be 10% with respect to the functional groups of ABS-G, was the same compared to ABS, indicating that graphene did not change the chemical structure of ABS [32]. Absorption peaks at 1489 and 1632 cm^−1^ belong to the nitrile group and double bonds of butadiene and double bonds of styrene in ABS polymers [33]. The absorption peak at 2920 cm^−1^ refers to the aliphatic and aromatic C–H bonds in ABS [34].

### 3.3. Electrochemical Characterization of 3D Printed ABS-G and ABS-G/ZVI Electrodes

**Figure 4:**
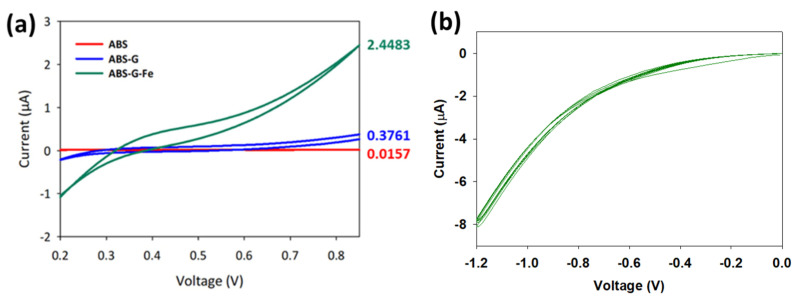
(**a**) CV curves of ABS, ABS-G, and ABS-G with electrodeposited ZVI; (**b**) electrodeposition of ZVI for five scan cycles using CV mode.

Figure 4a shows the CV curves of the 3D printed ABS, 3D printed ABS-G, and 3D printed ABS-G with electrodeposited ZVI electrodes measured in 0.05 M K_3_[Fe(CN)_6_ solution at a scan rate of 0.1 V/s tested within the potential range of 0.2 to 1.0 V (vs. Ag/AgCl). With just ABS, negligible electron-transfer capability was observed, indicating the inability of the ABS polymer to perform electrochemistry, whereas conductive ABS exhibited enhanced current detection. This was attributed to the graphene filler incorporation, which conferred the attractive properties of graphene, which has a single atomic layer of 2D carbon and exhibits excellent electron mobility, and graphite, with few or more layers of graphene. The electron mobility of graphene in ABS allows the material to be utilized as a transducer for sensor fabrication. Despite the ABS-G electrode depicting the capability of electron transfer, the conductivity was not feasible to be utilized directly in electrochemical sensing [35,36]. Thus, electrodeposition of ZVI onto 3D printed ABS-G was conducted to further enhance the redox capability of the 3D electrode, corresponding to 0.05 M ferricyanide as the electrolyte, as can be seen in Figure 4.

The ZVI suspension was electrodeposited onto the substrate 3D printed ABS-G electrode as shown in Figure 4b for five scan cycles. The electrodeposition process was performed in CV mode, with a running voltage from 0 V to −1.2 V for five scan cycles. During the process, a thin layer of ZVI was electrochemically deposited onto the 3D printed ABS-G electrode surface as shown in the FE-SEM and EDX images in Figure 2b. Upon successful completion of the electrodeposition process, the 3D printed ABG-G/ZVI electrode was fabricated and ready for application.

### 3.4. Calibration in 3-MCPD Using ZVI-3D Printed ABS-G Electrode

In order to test the performance of the 3D printed ABS-G electrodeposited with ZVI (denoted as ABS-G/ZVI electrode) in detecting 3-MCPD, calibration was performed using amperometric method. The calibration was conducted in 0.3 M NaOH solution at a fixed potential of 1.3 V, with the addition of 10 µL of 3-MCPD standard suspension at 300 s time intervals. The detection of 3-MCPD was achieved through a combination of surface adsorption and electron transfer happening in the redox solution, resulting in the current output measured.

**Figure 5:**
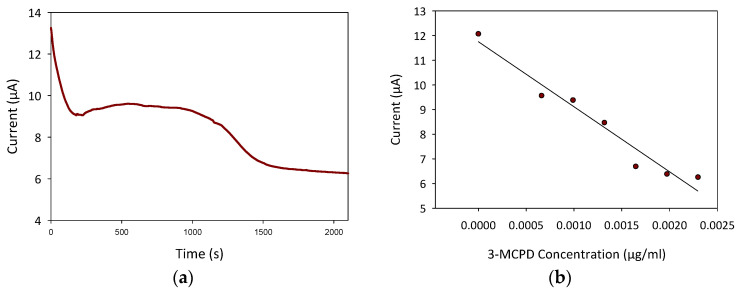
(**a**) Amperometric curves of ABS, ABS-G, and ABS-G with electrodeposited ZVI; (**b**) calibration plot of current measured versus 3-MCPD concentration (*R^2^* = 0.9539).

As shown in Figure 5a, at t = 0, the current measured was due to the movement of electrons in 0.3 M NaOH. Upon the first addition of 10 µL of 3-MCPD at t = 300 s, the redox peak current started to change direction, possibly due to the adjustment of 3-MCPD in the NaOH solution. Further addition of 10 µL of 3-MPCD at t = 600 s resulted in a decrease in the peak current as the 3-MCPD concentration increased. This decreasing peak current trend showed that the 3D printed ABS-G/ZVI sensor successfully detected 3-MCPD in the 0.3 M NaOH, with similar trends reported by [33,34,37]. It is important to determine the incubation time between each addition of 3-MCPD standard suspension prior to performing amperometry to ensure a more reflective and better peak current measured. The incubation time for each experiment varies according to the setting. For instance, 3-MCPD detection using imprinted *p*-aminothiophenol (*p*-ATP-AuNP/GCE) in soy sauce requires at least 6 min incubation time before the measurement of peak current using the DPV method [38]. The calibration plot of current measured according to the corresponding 3-MCPD concentration (*R^2^* of 0.9539) in Figure 5b exhibits a linear response range from 6.61 × 10^−4^ to 2.30 × 10^−3^ µg/mL with an estimated limit of detection of 3.30 × 10^−4^ µg/mL against 3-MCPD detection. A comparison of past reports related to electrochemical detection of 3-MCPD mostly in soy sauce can be seen in Table 1 below. Most past reports utilized gold nanomaterials and molecularly imprinted polymers (MIP) to enhance the sensitivity and selectivity of the fabricated sensor in detection 3-MCPD against other analytes. None of the studies utilized electrodeposited ZVI as the transducer nanomaterial, and none of the studies reported on 3-MCPD sensing in edible oils.

The preliminary results reveal a promising alternative platform for 3-MCPD detection, which can be performed outside the laboratory, with cheaper and faster results compared to the current method using GC–MS. GC–MS requires trained personnel; therefore, an alternative method that is simpler and more user-friendly should be developed, as the identification of 3-MCPD is currently attracting great attention. Furthermore, due to the complexity of 3-MCPD that can appear in both esterified and free forms, further electrode modification is needed in order to improve the detection sensitivity and selectivity against other contaminants, especially when tested in a complex environment such as edible oil. Depending on the target application, the detection and measurement of 3-MCPD in free or esterified form might require further sample preparation to be performed prior to the detection of 3-MCPD as the targeted analyte. For instance, an indirect traditional method of detecting 3-MCPD using GC–MS requires the sample to undergo transesterification to release 3-MCPD from its esterified form. Therefore, if the target is to detect and measure total 3-MCPD content, a similar transesterification process might be required prior to electrochemical analysis.

One of the improvements that can be achieved for this 3D printed electrode for 3-MCPD detection is related to its stability against harsh and complex environments, e.g., when testing in hot edible oil. Improvements must also be made on the sensitivity of the sensor by manipulating a nanocomposite of ZVI and polymers as the transducer layer. This can also help to preserve the shelf-life of the sensor for storage purposes. In a nutshell, developing a rapid test kit (RTK) for 3-MCPD detection in households represents a future direction in electrochemical sensing and should be made accessible to all, affordable, and easy to use.

## 4. Conclusions

The recent advancement in 3D printing technology for sensor fabrication has enabled analyte detection in various environments using electrochemical sensing, which is a simpler and cheaper production method. The 3D printing approach was applied by incorporating conductive fillers in the form of graphene into commercially available polymers of ABS. Successful adhesion of ZVI nanomaterial was also performed using the electrodeposition method, which further enhanced the redox capability of the tailored design 3D printed electrode. In this study, a 3D printed electrode using ABS with functionalized graphene was fabricated and tested for ZVI nanomaterial adhesion and 3-MCPD detection as the primary transducer element for future modification in electrochemical sensing applications, e.g., rapid and on-site detection of 3-MCPD in its free form. Further research to improve the stability, sensitivity, and selectivity of the fabricated 3D printed electrode to detect 3-MCPD in real life has to be achieved by improving the transducer and detection layers of the electrode sensor. The utilization of a nanocomposite with polymers can also help to enhance the 3-MCPD detection limit and selectivity. Successful calibration against 3-MCPD showed a promising opportunity in detecting this processed food contaminant, paving the way toward fabricating an electrochemical sensor with rapid, portable, and cheap sensing capabilities for 3-MCPD as the targeted analyte. Developing a 3-MCPD RTK for households can also help to improve the awareness of food contaminants and reduce the health risk of consuming food with contaminants above the tolerable daily intake (TDI).

## Figures and Tables

**Table 1:** Comparison between electrochemical 3-MCPD sensors based on past reports.

Electrode	Method of Detection	Linear Range	Lower Detection Limit	Reference
Glassy carbon electrode (GCE)/nanoporous gold (NPG)/molecularly imprinted polymer (MIP)	Differential pulse voltammetry (DPV)	10^−16^ to 10^−7^ mol/L	3.5 × 10^−17^ mol/L	[39]
Glassy carbon electrode (GCE)/hemoglobin immobilized with magnetic molecularly imprinted nanoparticles (Hb-MMIPs NPs)	Differential pulse voltammetry (DPV)	1.0 to 160.0 mg/L	0.25 mg/L	[40]
Gold (Au)/cysteine-coated silver nanoparticles (Cys-AgNPs)	Differential pulse voltammetry (DPV)	2.5 to 200 ng/mL	2.4 ng/mL	[41]
Glassy carbon electrode (GCE)/carboxylated multi-wall carbon nanotubes cMWCNT)/metal–organic framework (MOF-199)	Differential pulse voltammetry (DPV)	1.0 x 10^−9^ to 1.0 × 10^−5^ mol/L	4.3 × 10^−10^ mol/L	[37]
GCE/AuN/p-ATP	Differential pulse voltammetry (DPV)	1.0 x 10^−17^ to 1.0 × 10^−13^ mol/L	3.8 × 10^−18^ mol/L	[38]
